# The Role of Oxidative Stress-Related Gene Polymorphisms (*SOD2*, *GPX1*) in Severe Early Childhood Caries (S-ECC)

**DOI:** 10.3390/medicina61030432

**Published:** 2025-02-28

**Authors:** Timea Dakó, Ana-Petra Lazăr, Luminița Lazăr, Alexandra-Mihaela Stoica, Adriana-Stela Crișan, Monica Monea, Cristina-Ioana Bica

**Affiliations:** 1 Department of Odontology and Oral Pathology, George Emil Palade University of Medicine, Pharmacy, Science, and Technology of Targu Mures, Gh Marinescu 38, 540142 Targu Mures, Romania; timea.dako@umfst.ro (T.D.); alexandra.stoica@umfst.ro (A.-M.S.); monica.monea@umfst.ro (M.M.); 2 Department of Oral Rehabilitation and Occusology, George Emil Palade University of Medicine, Pharmacy, Science, and Technology of Targu Mures, Gh Marinescu 38, 540142 Targu Mures, Romania; 3 Department of Periodontology, George Emil Palade University of Medicine, Pharmacy, Science, and Technology of Targu Mures, Gh Marinescu 38, 540142 Targu Mures, Romania; luminita.lazar@umfst.ro; 4 Department of Genetics, George Emil Palade University of Medicine, Pharmacy, Science, and Technology of Targu Mures, Gh Marinescu 38, 540142 Targu Mures, Romania; adriana.crisan@umfst.ro; 5 Department of Pedodontics, George Emil Palade University of Medicine, Pharmacy, Science, and Technology of Targu Mures, Gh Marinescu 38, 540142 Targu Mures, Romania; cristina.bica@umfst.ro

**Keywords:** severe early childhood caries, oxidative stress, single nucleotide polymorphism, DMFT

## Abstract

*Background and Objectives*: Severe early childhood caries (S-ECC) is a chronic infectious disease with a multifactorial etiology which has not been completely elucidated. Research on the role of oxidative stress in the etiopathogenesis of oral diseases suggests that the level of local antioxidants plays an important role in determining susceptibility to caries. This study aimed to demonstrate that the host’s redox imbalance, modified by genetic polymorphisms, may influence the onset and severity of S-ECC. *Materials and Methods*: A total of 110 patients were included in the study (59 diagnosed with S-ECC and 51 healthy controls). Upon initial appraisal, the DMFT (decayed-missing-filled teeth) index was determined, and epithelial cells were collected using oral swabs for genomic DNA extraction. Genotyping of *SOD2* (rs4880) and *GPX1* (rs1050450) was performed using TaqMan SNP genotyping assays and real-time polymerase chain reaction (PCR). *Results:* According to the results of the present study, there was a significant difference between the frequency of the reference genotype and variants for *rs4880* (*p* = 0.0303). Subjects carrying the AG and GG variant genotype of *rs4880* were significantly associated with a high DMFT value (*p* = 0.0005). However, no significant difference was found between the genotypes for *rs1050450*, nor was there an association with the severity of S-ECC. *Conclusions:* The AG and GG variant genotypes of the *SOD2* polymorphism (rs4880) increase the severity of caries in preschoolers and predispose patients to develop carious lesions, especially when associated with certain feeding practices and infrequent toothbrushing. This observation emphasizes that host sensitivity to caries is a crucial factor in the onset and development of carious lesions in primary dentition, despite the main contributing factors to this pathology. The *rs1050450* polymorphism was not associated with the severity of S-ECC.

## 1. Introduction

The American Academy of Pediatric Dentistry introduced the term of “severe early childhood caries” (S-ECC) to refer to the clinical features of severe caries in the primary dentition, which have an atypical, progressive pattern and begin at a very young age [1]. The most important determinants for the onset and progression of dental caries in deciduous teeth are the host (tooth surface and saliva), microorganisms found in the oral cavity, fermentable carbohydrates, and time. In the development of the disease, the protective function of saliva—expressed through certain defense mechanisms against bacterial colonization activity and dental biofilm formation—is crucial. Saliva serves as a cleanser, buffer, and lubricant, and it also stores calcium and phosphate while continuously cleansing the teeth and oral mucosa [2,3,4].

Antioxidants (AO) present in saliva (like those in plasma and tissues) provide protection against free radicals (FR) and/or reactive oxygen species (ROS). These unstable molecules have increased reactivity because they contain a free electron that combines with various cellular components, reactions that cause DNA damage, mitochondrial dysfunction, and cell wall or cell destruction. Oxidative stress (OS), resulting from the imbalance between FR/ROS and AO, therefore involves the adverse effects of oxygen and other FR on living tissues. Within the salivary bioenvironment, OS is an important etiological factor in the occurrence of dental caries and oral inflammations [3]. By stimulating an inflammatory context that favors bacterial growth and tissue deterioration, OS is one of the key metabolic causes underlying oral pathologies, including dental decay. Furthermore, it worsens oral pathologies by aggravating tooth surface damage and increasing tissue inflammation in rampant caries cases, such as S-ECC. Excessive ROS production damages the oral cavity’s lipids, proteins, and DNA, compromising the structural integrity of dental tissues and increasing their susceptibility to acid attack by cariogenic bacteria [4,5].

The level of AO in saliva depends on very few components, including the peroxidase enzyme and the uric acid molecule. The peroxidase enzyme system has been found to possess significant antistreptococcal properties, inhibiting the growth of cariogenic bacteria by catalyzing the peroxidation of the thiocyanate ion (SCN^−^) into a more stable oxidation product (OSCN^−^). Antioxidants like glutathione peroxidase (GPx), catalase, and superoxide dismutase (SOD) eliminate ROS in a healthy system, preserving the redox balance [3,6]. The relationship between S-ECC and the genetic coding of the various oxidative stress-related genes involved is lacking conclusive evidence in the pediatric literature. Genetic factors influence oxidative stress within S-ECC through the fact that polymorphisms of genes encoding specific antioxidant enzymes, which are found in saliva, create a predisposition to an increased level of salivary oxidative stress, creating a favorable environment for the onset of caries on deciduous teeth [7].

The polymorphisms of genes encoding the manganese superoxide dismutase (MnSOD) and glutathione peroxidase 1 (GPx1) enzymes are positively associated with harmful modifications in OS. The polymorphism encoding MnSOD, located in the *SOD2* gene, especially Val16Ala, significantly reduces enzymatic activity and facilitates the accumulation of ROS, exacerbating OS in hard dental structures and increasing susceptibility to the onset of carious lesions. Through alterations in enzyme location and mitochondrial transport, the polymorphism of the manganese *SOD2* gene (rs4880) impacts the redox status balance [8,9]. Nucleotide substitution (T, thymine → C, cytosine) and successive amino acid substitution of alanine (Ala) with valine (Val) (Ala16Val) comprise the single nucleotide polymorphism (SNP) in the *SOD2* gene (rs4880), which ultimately leads to a 30–40% reduction in the efficiency of *SOD2* Val allele transport in the mitochondria and a decreased capacity to neutralize superoxide anions. Additionally, environmental factors further influence the *SOD2* SNP [10,11,12,13,14].

Furthermore, SNPs which are associated with non-enzymatic systems, such as those encoding glutathione S-transferase, also play an active role in the mechanism of oxidative stress. Through the reaction catalyzed by GPx1 and other peroxidases, glutathione neutralizes free radicals either directly or indirectly, including nitric oxide and H_2_O_2_. Nucleotide substitution (C, cytosine → T, thymine) and the successive replacement of the amino acid proline (Pro) with leucine (Leu) are characteristics of the *GPX1* (rs1050450) gene polymorphism. In contrast to the Pro allele, the *GPX1* C593T variation is thought to cause decreased mRNA expression and enzyme activity in the presence of the Leu allele [9,14,15].

The prevalence of S-ECC, a chronic infectious disease caused by cariogenic bacteria and facilitated by frequent contact of tooth surfaces with carbohydrates from milk and fruit juices, ranges from 7 to 50% worldwide in children under the age of six, and from 15 to 18% in Romania [2]. The high prevalence of children with S-ECC and the increased values of carious experience indices identified in preschool children in Romania indicate the need for a concentrated effort to reduce the incidence and severity of the disease [4]. Children with dental caries in general have been proven to have a unique salivary antioxidant response to OS, as seen by increased levels of SOD and total antioxidant capacity. It is essential to implement prevention programs for this condition that include both local oral hygiene and prophylaxis measures, as well as general advice to parents regarding the effect of carbohydrates on the etiopathogenesis of dental caries. The relationship between biological antioxidants and nutrition should also be addressed [16,17]. This justifies why it is crucial to identify children presenting genetic mutations of enzymes and non-enzymatic systems which control OS at very young ages. Children are the most vulnerable members of the demographics and are more prone to acquire mutations that ultimately lead to the development of oral conditions caused by the redox state imbalance [18,19].

The null hypothesis (H0) of this study was that there is no link between the DMFT index of children diagnosed with S-ECC and the *SOD2* and *GPX1* gene variations. The aim of this study was to investigate the SNPs in *SOD2* and *GPX1* genes which are implicated in OS and to analyze the association between the allele variants of *SOD2* (rs4880) and *GPX1* (rs1050450) SNPs and the predisposition to S-ECC, in a Romanian population. This research also aimed to verify how the DMFT (decayed-missing-filled teeth) score varies depending on the age and gender of children diagnosed with S-ECC.

## 2. Materials and Methods

### 2.1. Ethical Considerations

The study was undertaken in accordance with the Declaration of Helsinki after receiving the approval from the Scientific Research Ethics Committee of G. E. Palade UMPhST of Targu Mures no. 2424/04.07.2023. Before the study commenced, written authorizations and consents concerning genetic testing and oral examinations were obtained from participating parents and caregivers of the children.

### 2.2. Study Population

This research was conducted as a single-center observational prospective study, between 1 March 2024 and 30 June 2024. The analysis included 110 consecutive patients who came to the Pedodontics Clinic of the Dental Medicine Faculty and met the inclusion criteria (Table 1).

### 2.3. Clinical Examination

The clinical examination and DMFT calculations were performed by two calibrated dentists in the Pedodontics Department of the Faculty of Dental Medicine, G. E. Palade UMPhST of Targu Mures. The examination was performed under natural light using dental mirrors and explorers.

Carious experience was established by calculating the DMFT (decayed-missing-filled teeth) index which sums up all the carious/absent/filled teeth in the oral cavity. The DMFT index values range from 0 (subjects without caries) to 20. A DMFT score of 4 or higher by the age of three years, DMFT ≥ 5 at 4 years, and DMFT ≥ 6 at 5 years establish the diagnosis of S-ECC [1]. In children without carious lesions, the DMFT score is equal to 0 (Table 2). Children with typical caries patterns were excluded from this study. The patients were then divided into two groups: the study group with 59 patients diagnosed with S-ECC, and the control group with 51 patients with no carious lesions (DMFT = 0). The children’s age, gender, residential background, dietary habits (feeding practices, snack preferences, frequency of sugar consumption between meals), oral hygiene practices (frequency of toothbrushing, usage of fluoride toothpaste), and frequency of dental checkups were also recorded.

### 2.4. Sample Collection and Genetic Analysis

Epithelial cells from the oral mucosa were collected utilizing Isohelix SK-3S Buccal Swabs, which were ethylene oxide-treated (Isohelix Ltd., Kent, UK). The swab was inserted into the oral cavity of the subjects and rubbed firmly against the buccal mucosa and underneath the lower lip for 60 s. The swab was then inserted into a 2 mL Eppendorf safe-lock tube containing 1 mL of phosphate-buffered saline (PBS). Each tube was coded due to confidentiality reasons and transported to the Molecular Biology/Genetic Laboratory of the Center for Advanced Medical and Pharmaceutical Research of G. E. Palade UMPhST of Targu Mures to perform DNA isolation and genotyping.

For the DNA isolation step, the PureLink Genomic DNA kit (Thermo Fisher Scientific, Carlsbad, CA, USA) was used following the manufacturer’s protocol. DNA quantification was then performed using an Eppendorf BioSpectrometer (Eppendorf, Wien, Austria GmbH). For *SOD2 rs4880* and *GPX1 rs1050450* genotyping, we used TaqMan assays (C_8709053 and C_175686987_10, Thermo Fisher Scientific, Carlsbad, CA, USA) and 7500 Fast Dx Real-Time PCR system (Applied Biosystems, Foster City, CA, USA).

### 2.5. Statistical Analysis

All data were collected in Microsoft Excel worksheets (Microsoft Corporation, Washington DC, USA, 2018). The statistical analysis was performed in GraphPad Prism version 10.2.3 for Windows (GraphPad Software, San Diego, CA, USA). A priori power analysis was conducted for the chi-square test, assuming a medium effect size (w = 0.3) and a significance level of 0.05. The analysis indicated a statistical power of 0.88, confirming that the study was well-powered to detect medium-sized effects. Firstly, descriptive statistics such as mean and standard deviation were assessed. Data normality was determined using the Kolmogorov–Smirnov test. The chi-square test was used for determining the difference between the reference genotype and variant genotypes for both SNPs in patients and controls. The Kruskal–Wallis test was used to investigate the difference between the DMFT index among patients with the homozygous reference genotype and heterozygous or homozygous variants of SNPs. Post-hoc pairwise comparisons were performed using the Dunn test to determine which specific groups differed significantly from each other. Univariate regression was performed to determine the predictor genotype variant for S-ECC. Relative risk (RR) was calculated to quantify the strength of the studied genotypes. The confidence level (CI) was established at 95%.

## 3. Results

**Figure 1:**
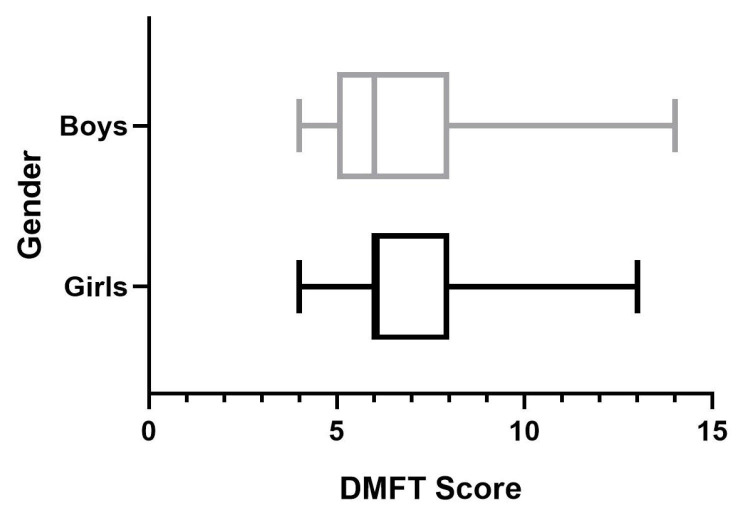
DMFT score distribution based on gender.

The average DMFT score was slightly higher for the group of boys (7.33) compared to the girls (7.13). The DMFT distribution for boys and girls is presented in Figure 1.

**Figure 2:**
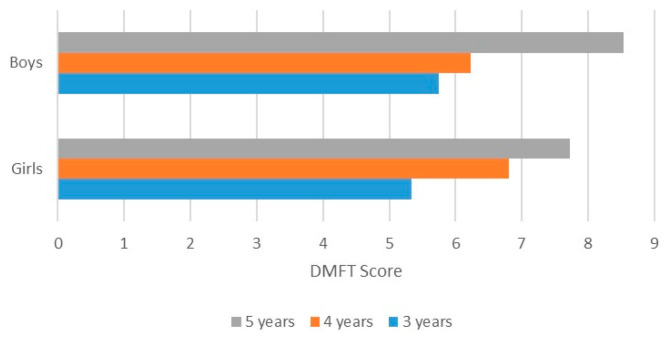
DMFT score distribution based on gender and age.

When calculating the DMFT values by age groups, no statistically significant difference was found between boys and girls (age 3: *p* = 0.0823, age 4: *p* = 0.2675, age 5, *p* = 0.3134). The DMFT index increases proportionally with age, the average being 6.60 for the age of 3, 7.14 for the age of 4, and 7.87 for the age of 5. Its distribution for both girls and boys are represented in Figure 2.

Table 3 summarizes the findings regarding the reference and variant genotype distribution of the two polymorphisms (rs4880 and rs1050450).

A significant difference was found between the genotype frequency for patients and controls for the *SOD2* polymorphism (*p* = 0.0303, RR = 0.6317). The AA reference genotype of the *SOD2* gene, which is associated with a high MnSOD activity, and is considered protective against oxidative stress, was more frequent in controls than in patients with S-ECC. The AG and GG variants, which have moderate and low enzymatic activity, were more frequent in patients with caries than in controls. No statistically significant difference between alleles for the *rs4880* polymorphism (*p* = 0.2098) were detected.

**Figure 3:**
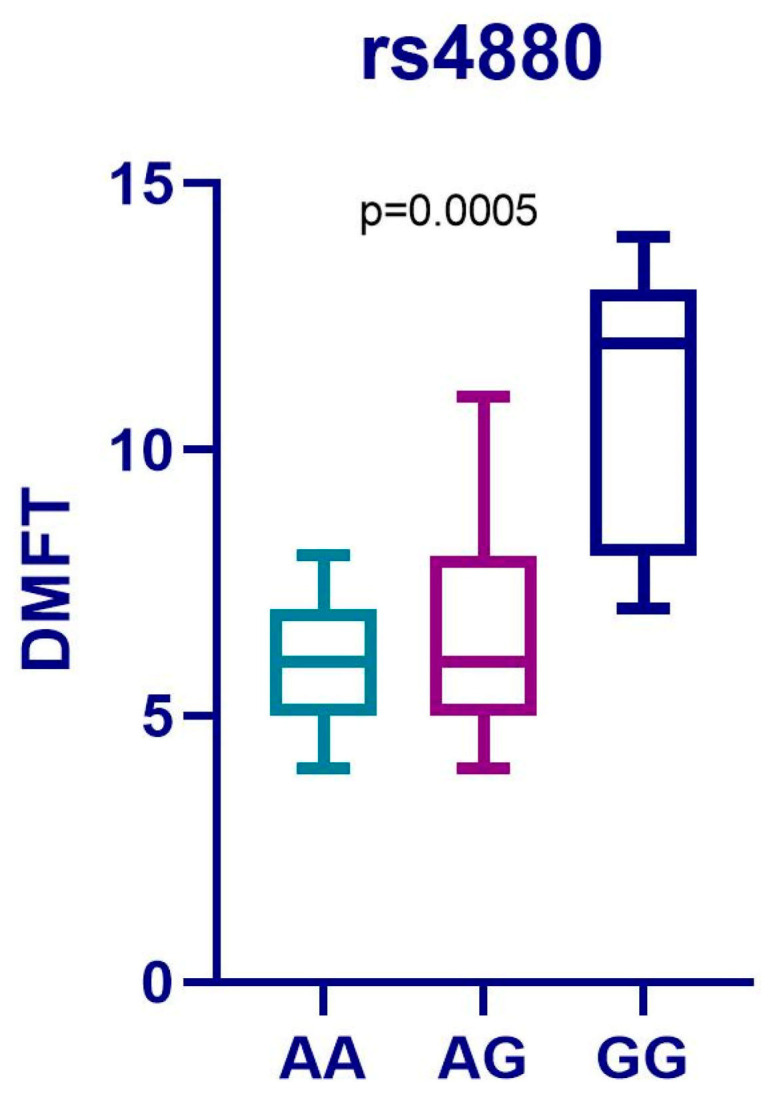
Results of the Kruskal–Wallis test for rs4880.

A statistically significant difference between the DMFT values of the patients with the reference genotype and the two variants (*p* = 0.0005) was determined (Figure 3).

The Post-Hoc Dunn’s test using a Bonferroni corrected alpha of 0.017 indicated that the mean ranks of the following pairs are significantly different: AA-GG, AG-GG. In the univariate regression analysis, the presence of the GG variant of the *rs4880* polymorphism was an independent predictor for the presence of S-ECC and its severity (*p* = 0.03, RR = 2.62, 95% CI, 1.10–6.23).

**Figure 4:**
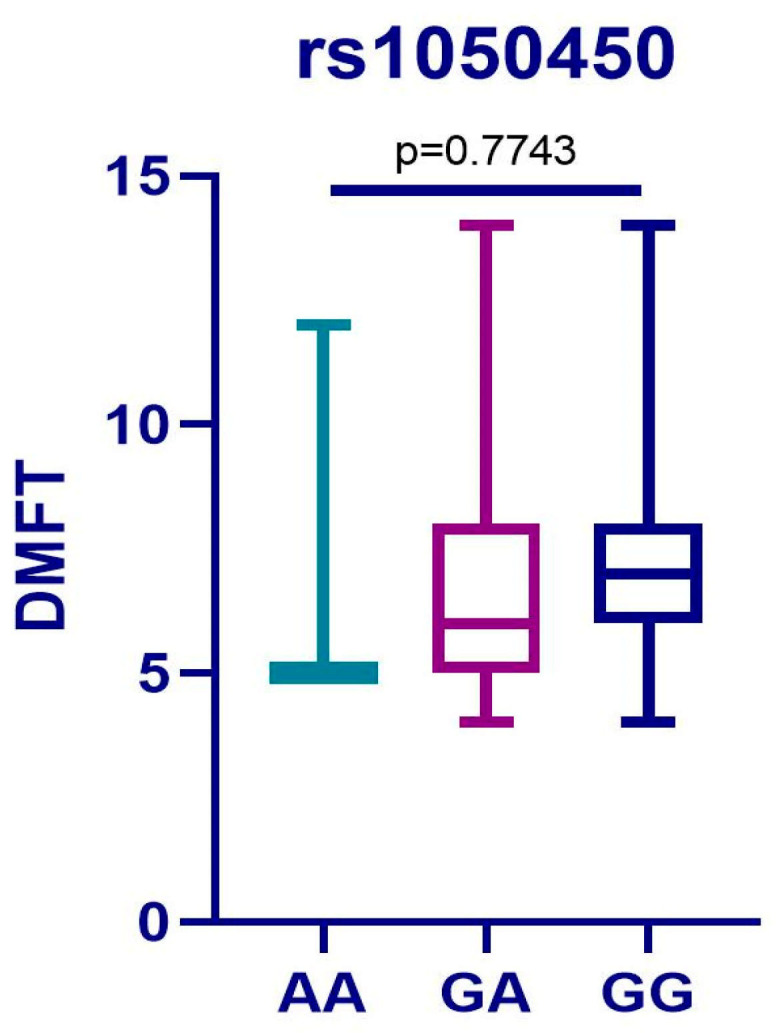
Result of the Kruskal–Wallis test for rs1050450.

Regarding the *GPX1* polymorphism, there was no statistically significant difference between the GG reference genotype which is protective against enzymatic activity and the AG or AA variants which have moderate and weak antioxidant defense function (*p* = 0.9889). Also, there was no statistically significant difference between the A and G alleles for the *GPX1* polymorphism (*p* = 0.6928). No statistically significant difference between the DMFT score of the patients with the reference genotype and variants for the *rs1050450* polymorphism (*p* = 0.7743) were found (Figure 4).

The dietary habits, oral hygiene practices, and residential background in relation to *SOD2* and *GPX1* polymorphism genotypes are presented in Table 4. No significant difference in the distribution of genotypes following the rs4880 and rs1050450 polymorphisms were observed, except two characteristics: (1) usage of fluoride toothpaste for the SOD2 polymorphism (*p* = 0.0131) and (2) frequency of brushing for the GPX1 polymorphism (*p* = 0.0459).

Since a significant difference in genotype frequency (reference and variant) between patients and controls for the *SOD2* polymorphism was observed, we aimed to investigate whether individuals with the variant genotypes also exhibited differences in dietary habits, oral hygiene practices and residential background between the two groups (patients and controls) (Table 5).

## 4. Discussion

Considering the limited research on the genetic involvement of OS-related enzyme gene polymorphisms, the present study assessed the role of the variant genotypes of rs4880 and rs1040450 and their association with the rampant caries pattern of S-ECC. This study also aimed to analyze the characteristics of the carious experience of the children included in the study, and how the DMFT score varied depending on age and gender.

The results of the present study showed a slight difference in the average DMFT score between boys and girls, with a minor value of 0.2 in favor of the boys group. Although the difference of the findings was negligible, it is in accordance with other studies that researched the prevalence of carious lesions in preschool children, in Romania and worldwide, where boys exhibited higher DMFT scores compared to girls [20,21,22]. No statistically significant difference between the mean DMFT score for boys and girls across the age groups of 3, 4, and 5 years was observed, although, for the age of 3 the difference had a trend towards statistical significance (*p* = 0.0823).

Environmental and genetic variables, microbiological factors, dietary habits, fluoride exposure, oral hygiene, saliva composition, and tooth structure all influenced the development of caries.

For the preschool child’s wellbeing, it is crucial to keep the primary dentition in good condition. Early restorations, nutrition counseling, parent education on decay-promoting feeding practices, maintaining proper dental hygiene, and using preventive medicines like topical fluorides can all help stop the progression of the S-ECC [4,23]. The treatment of S-ECC is high-priced and frequently necessitates early tooth extraction and major restorative procedures. Occasionally, deep sedation or general anesthesia is necessary because young children are unable to handle the complex and long treatment procedures [24,25].

Early diagnosis of S-ECC can be improved by identifying salivary biomarkers, gene polymorphisms of OS-related genes, and microbiome changes that indicate enamel demineralization. Risk assessment models incorporating genetic susceptibility, dietary habits, and socio-behavioral factors can help classify children into high-, moderate-, and low-risk groups for targeted prevention [13]. Regular dental checkups, beginning in infancy at the age of 1, allow for early detection of subtle enamel demineralization and timely intervention before decay progression. Preventive strategies such as fluoride application, dietary modifications, and caregiver education can significantly reduce the prevalence of S-ECC, especially in high-risk populations. Integrating personalized preventive care plans and community-based programs can enhance long-term oral health outcomes and minimize the burden of S-ECC in preschool children [24].

Although the sample size of this study was small and the results could only be limitedly generalized, they can be considered for future research directions that may lay the foundations of a strong oral health prevention program in Romania. The high DMFT scores registered in these preschool children suggests that a general effort is needed to implement effective prevention programs from both clinicians, the Romanian healthcare system, and parents of the children [26].

The enzymatic antioxidant system, which includes proteins with enzymatic antioxidative abilities that are encoded by matching genes, controls the effective elimination of ROS. The activity of important antioxidant enzymes can be modified by SNPs in those genes, which can lead to imbalances in the oxido-redox state of cells [7,27]. Certain genetic variations can affect a person’s vulnerability to environmental influences and predispose them to the onset and severity of specific diseases. This applies to dental caries in general, and specifically for S-ECC, as genetic predisposition to low or moderate OS enzymatic activity, combined with environmental factors may create an optimal environment for rampant caries.

The Ala16Val polymorphism has been identified in exon 2 of the human SOD2 gene, which is encoded by the nuclear *SOD2* gene found on chromosome 6q25. An often-researched *SOD2* SNP on the *SOD2* gene, Ala16Val (47C>T or rs4880) is known to result in a conformational change. The mitochondrial targeting sequence contains the *SOD2* Ala16Val polymorphism, which has been known to alter the peptide structure and impact protein maturation and translocation into the mitochondrial matrix. This polymorphism has not been linked to the onset and development of dental caries to date [6,28,29].

Our study demonstrated that there was a significant difference between the genotype frequency of patients and controls for the rs4880 polymorphism (*p* = 0.0303). This suggests that carrying at least one A allele may be protective against the disease, potentially following a dominant model of inheritance. The patients who had homozygous (GG) or heterozygous (AG) variant genotypes were significantly associated with higher DMFT scores than those with the reference genotype (AA) (*p* = 0.0005). This implies that the variant genotypes influence the severity of caries in S-ECC in a detrimental way. However, there was no statistically significant difference between the allele frequency for *rs4880*, which may be caused by the small sample size.

Other studies have demonstrated a positive correlation between the severity of caries and SOD2 activity, which was found to be considerably higher in the saliva of caries-active patients than in healthy controls [5]. Furthermore, a prior study demonstrated that oxidative stress-induced impaired ameloblast and enamel matrix maturation stages are linked to dental fluorosis (DF) as well, which is a noncariogenic disease of the dental hard tissues [30]. Researchers have proposed that the mechanism of fluoride-induced ameloblast apoptosis also involves *SOD* activity [31]. A recent cross-sectional study found that polymorphisms in the *SOD2* gene, such as rs10370, rs4880, and rs5746136, were significantly linked to the risk of developing DF. This is likely because *SOD2* gene mutations result in impaired ROS scavenging, oxidative stress damage, and the apoptosis of cells that form enamel [30]. The univariate regression has demonstrated the presumable role of the *rs4880* SNP in being linked to the severity of S-ECC, within the limitations of our research.

The *GPX1* gene is located on chromosome 3p21.3 and consists of two exons with a 1.42 kb area [16]. A genetic variation at codon 198 of the *GPX1* gene (198Pro/leu, rs1050450) results in the substitution of leucine (CTC) for proline (CCC). The GPX1 198Leu allele was linked to a markedly lower *GPX1* enzymatic activity than the 198Pro allele, according to recent research [17,32].

The results regarding rs1050450 polymorphism (*GPX1*) were not of important connotation as the allele frequency (*p* = 0.6928) and genotype association (*p* = 0.7743) with the severity of caries have not reached statistical significance. The wild-type genotype (GG) of *GPX1* is associated with optimal antioxidant capability and greater efficiency in neutralizing ROS. However, the variant genotypes (GA and AA) exhibit low to moderate antioxidant capacity, having a significant biological implication in diseases where OS plays an important role. This outcome highlights the complexity of genetic implications in the etiology of S-ECC.

Though this study may have been limited by its small sample size, further studies on larger patient groups may demonstrate that the rs1050450 polymorphism increases caries susceptibility by maintaining a high ROS level in the saliva and weakening the surface of the enamel. With a compromised antioxidant system and favorable environmental factors, such as poor oral hygiene, a sugary diet, and the lack of preventive measures, *GPX1* could increase the susceptibility of preschool children to S-ECC [17,32]. An OS-related gene polymorphism (such the rs1050450) may not be significantly linked to caries susceptibility on its own, but if associated with other gene polymorphisms, it may raise the risk of caries. Thus, the authors suggest that future research should explore the combined impact of haplotypes and gene polymorphisms in the etiopathogenesis of S-ECC [33]. Genetic profiling could help identify individuals at a higher risk of developing S-ECC. Determining the gene polymorphisms of OS-related genes in S-ECC holds significant clinical relevance, particularly for understanding the genetic susceptibility to dental decay and guiding personalized prevention and treatment strategies [34,35]. Rapid tests or other easily accessible methods could potentially be developed to identify children who are more susceptible to this condition based on genetic and other biological markers. Saliva-based tests could be developed to detect specific polymorphisms of OS-related genes and enamel formation genes, both of which are associated with an increased risk of S-ECC. As these tests are non-invasive, quick, and simple to perform, they could provide a fast way to assess genetic susceptibility. A multifactorial risk assessment tool could also be helpful, by providing a comprehensive evaluation of multiple factors, such as genetics, oral hygiene habits, diet, and family history. By combining fast genetic testing with lifestyle and environmental factors, it could create a personalized risk profile for preschool children [36].

The authors’ findings revealed a statistically significant difference in fluoride toothpaste use between individuals carrying the AG+GG variants and those with the AA genotype of *SOD2* (rs4880). This result suggests that genetic variability in SOD2 may influence oral hygiene habits or fluoride sensitivity. The results after analyzing whether the AG and GG variants of rs4880 predispose patients to develop S-ECC or exacerbate its severity, suggest that while most dietary habits did not differ between patients and controls, variations in feeding practices and the frequency of toothbrushing may play a role in the observed differences, potentially influencing disease outcomes. Bottle or combined feeding habits and irregular toothbrushing contribute to symptom deterioration for patients who carry the variant genotypes of the *SOD2* SNP. These findings are not unexpected, given the multifactorial etiology of S-ECC. Further investigations are necessary to elucidate whether this association is mediated by behavioral adaptation or an inherent biological response to fluoride exposure. Similarly, for *GPX1* (rs1050450), we observed a significant difference in toothbrushing frequency between individuals with the GA+AA variants and those with the GG genotype. This genetic variation could be linked to differences in salivary antioxidant capacity, indirectly influencing oral hygiene habits.

These findings underscore the potential interplay between genetic predisposition to oxidative stress and oral hygiene behaviors. While behavioral factors are modifiable, underlying genetic susceptibilities may shape individual responses to oral health recommendations. Future research should explore whether these polymorphisms also influence clinical outcomes related to S-ECC and whether targeted preventive strategies could be tailored based on genetic profiles [29,32].

The authors acknowledge the relatively small sample size as a shortcoming of this study, as it may reduce the statistical power to detect a significant association. Another element of limitation was that this research did not explore the enzymatic activity or oxidative stress markers in the affected children. By advancing research in the salivary enzymatic activity, early detection of S-ECC, risk assessment, and personalized preventive strategies could be established through biochemical validation. An additional limitation of this study is the lack of assessment of dental plaque quantity and composition and other socio-economic factors, as well as their potential role in the development of S-ECC.

## 5. Conclusions

In this study, the authors concluded that the AG and GG variant genotypes of the *SOD2* gene (rs4880) predispose patients to develop carious lesions and influence the severity of caries in preschool children. Differences in feeding practices, like bottle or combined feeding habits, and infrequent toothbrushing could contribute to the observed variations and may affect disease outcomes for the carriers of the variant genotypes.

Rs1050450 does not show a statistically significant association with S-ECC in this patient sample.

To reduce the prevalence of dental caries, these findings highlight the importance of genetic polymorphisms, which enable clinicians to alert the parents and caregivers of the children about their risk of developing dental caries and to encourage better oral hygiene practices by explaining the host susceptibility. Furthermore, implementing oral health prevention projects may help mitigate the impact of genetic variations on the onset and development of S-ECC. Although there are many contributing factors to dental caries in primary dentition, this study highlights that host sensitivity to caries is a significant component.

## Figures and Tables

**Table 1:** Patient selection criteria.

Inclusion Criteria	Exclusion Criteria
Children aged 3–5 years Children with primary dentition	Children with normal caries pattern
Children with S-ECC caries pattern	Children with general or mental conditions, under specific medication
Children without carious lesions	Children with dental fluorosis and developmental hypoplasia Uncooperative children

**Table 2:** Classification of DMFT scores.

Age (Years)	S-ECC (Study Group)	Children with No Caries (Control Group)
3	DMFT ≥ 4	DMFT = 0
4	DMFT ≥ 5	DMFT = 0
5	DMFT ≥ 6	DMFT = 0

**Table 3:** Genotype and variants of *SOD2* and *GPX1* polymorphisms in the studied population.

Polymorphism	Patients (*n* = 59)	Controls (*n* = 51)	Statistical Analysis for Genotypes	Statistical Analysis for Alleles
*SOD2*—rs4880 A/G				
A/A	13	21	A/G+G/G vs. A/A *Chi-square* *p* = 0.0303 *, RR = 0.6317 95% CI: 0.38–0.96	G vs. A *p* = 0.2098 95% CI: 0.68–1.09
A/G	39	26		
G/G	7	4		
A	65	68		
G	53	34		
*GPX1*—rs1050450 G/A				
GG	29	25	G/A+A/A vs. G/G *Chi-square* *p* = 0.9889, RR = 1.002	A vs. G *p* = 0.6928 95% CI: 0.50–0.85
G/A	27	21		
A/A	3	5		
G	85	26	95% CI: 0.70–1.42	
A	33	31		

* Statistically significant.

**Table 4:** Patient genotype distribution based on dietary habits, oral hygiene practices, and residential background.

Characteristics	Patients (*n*)	*SOD2* (rs4880)			Patients (*n*)	*GPX1* (rs1050450)		
		AA	Variant AG+GG	*p*-Value		GG	Variant AG+AA	*p*-Value
**Feeding practices**								
Bottle	21	8	13	0.0719	12	5	7	0.0734
Breastfeeding	28	3	25		26	10	16	
Combined	10	2	8		21	14	7	
**Snacking preferences**								
Sweets	30	9	21	0.2163	14	6	8	0.2217
Fruits	21	2	19		34	15	19	
Salty snacks	8	2	6		11	8	3	
**Sugar consumption between meals**								
Daily	17	6	11	0.1640	19	12	7	0.2281
3–4 times/week	16	1	15		16	9	7	
1–2 times/week	21	4	17		19	6	13	
Once every few weeks	5	2	3		5	2	3	
**Frequency of brushing**								
Twice a day	17	4	13	0.7955	10	4	6	**0.0459** *
Once a day	19	5	14		23	8	15	
3–4 times/week	20	3	17		15	12	3	
Once a week	3	1	2		11	5	6	
**Fluoride toothpaste**								
Yes	44	6	38	**0.0131** *	28	11	17	0.2380
No	15	7	8		31	18	13	
**Frequency of dental checkups**								
Twice a year	15	2	13	0.7066	9	3	6	0.7244
Once a year	25	6	19		24	13	11	
Once every few years	6	1	5		17	9	8	
Never	13	4	9		9	4	5	
**Residential background**								
Rural	31	6	25	0.99	31	17	14	0.4379
Urban	28	7	21		28	12	16	

* Statistically significant.

**Table 5:** Variant genotype distribution for patients and controls based on dietary habits, oral hygiene practices, and residential background.

Characteristic	Total Patients for *SOD2* SNP (rs4880) (*n*)	Patients AG+GG	Controls AG+GG	*p*-Value
**Feeding practices**				
Bottle	26	13	13	0.0260 *
Breastfeeding	32	25	7	
Combined	18	8	10	
**Snacking preferences**				
Sweets	38	21	17	0.5377
Fruits	3	19	11	
Salty snacks	8	6	2	
**Sugar consumption between meals**				
Daily	20	11	9	0.7040
3-4 times/week	27	15	12	
Once every few weeks	26	17	9	
**Frequency of toothbrushing**				
Twice a day	17	13	4	0.05 *
Once a day	3	14	16	
3-4 times/week	23	17	6	
Once a week	6	2	4	
**Fluoride toothpaste**				
Yes	62	38	24	0.9873
No	14	8	6	
**Frequency of dental checkups**				
Twice a year	18	13	5	0.1676
Once a year	31	19	12	
Once every few years	14	5	9	
Never	13	9	4	
**Residential background**				
Rural	42	25	17	0.8425
Urban	34	21	13	

* Statistically significant. No significant differences were observed between patients and controls in dietary habits, except for feeding practices (*p* = 0.0260) and the frequency of toothbrushing (*p* = 0.05), which showed a notable variation between the groups.

## Data Availability

The original contributions presented in this study are included in the article. Further inquiries can be directed to the corresponding author, upon reasonable request.
